# Nomogram-based prediction of continued axial elongation in children undergoing orthokeratology

**DOI:** 10.3389/fmed.2025.1686204

**Published:** 2025-11-12

**Authors:** Xiangxiang Fu, Quanyong Yi, XiaoLi Mao, Shuaili Zhen, Fangfang Han, Qianwei Zhu, Zhenni Du, Xuchong Pan, Yiran Hu, Jianing Ying, Xiang Li, Yeshuang Wu

**Affiliations:** 1 Yuyao Maternity and Child Health Care Hospital (Yuyao Second People’s Hospital), Ningbo, China; 2 Ningbo Eye Hospital, Wenzhou Medical University, Ningbo, China

**Keywords:** myopia, orthokeratology, predictive model, risk stratification, personalized medicine

## Abstract

**Background:**

Myopia is a growing health concern, especially among children, with Orthokeratology (OK) lenses showing promising results in myopia control. However, treatment outcomes vary significantly among individuals, highlighting the need for personalized approaches. This study aimed to develop and validate a predictive model for OK therapy outcomes in myopic children.

**Methods:**

This retrospective cohort study included 439 myopic patients fitted with OK lenses. Patients were randomly divided into training (*n* = 308) and test (*n* = 131) sets. Least absolute shrinkage and selection operator regression was used for variable selection, followed by logistic regression to construct the predictive model. A nomogram was developed to visualize individual risk predictions. Model performance was assessed using calibration plots, receiver operating characteristic (ROC) curves, and decision curve analysis (DCA).

**Results:**

Four variables were identified as significant predictors: age, parental myopia, white-to-white distance, and spherical refraction. The model demonstrated good discriminatory ability with areas under the ROC curve of 0.831 (95% CI: 0.786–0.877) in the training set and 0.820 (95% CI: 0.742–0.899) in the test set. Sensitivity and specificity were 75.6 and 72.8% in the training set, and 79.3 and 75.0% in the test set. Calibration plots and DCA confirmed the model’s potential clinical utility across a range of threshold probabilities.

**Conclusion:**

This study developed a predictive model for OK therapy outcomes in myopic children. The model demonstrated good discriminatory ability in both training and test datasets. This predictive approach might contribute to risk stratification in myopia management. Further validation through prospective studies across diverse populations is needed before such models could potentially inform clinical decision-making and resource allocation in myopia control practice.

## Introduction

Myopia is a pervasive global health issue, with East Asia showing the highest prevalence rates globally, reaching 69% among 15-year-olds and up to 86% among Singaporean-Chinese children (1). The global prevalence of myopia increased from 24.32% in 1990 to 35.81% in 2023 and is projected to reach 39.80% by 2050, exceeding 740 million cases worldwide (2). High myopia is associated with serious ocular complications such as retinal detachment, macular degeneration, glaucoma, and cataracts, which can lead to irreversible vision loss (3, 4). These complications contribute not only to individual visual impairment but also to substantial economic and social burdens, particularly in regions with high myopia prevalence (5, 6). The increasing prevalence among younger populations, combined with the severe long-term consequences of myopia, underscores the urgent need for early intervention and effective management strategies (7). Controlling axial length (AL) elongation is a key strategy to mitigate these risks and reduce the impact of myopia (8, 9).

Orthokeratology (OK) lenses, which are rigid gas-permeable lenses worn overnight to temporarily reshape the cornea, have gained widespread acceptance as an effective intervention to slow AL elongation, particularly in children and adolescents (10, 11). Studies have shown that OK lenses can reduce annual AL growth by 59% compared to untreated controls (12). Their non-invasive nature and potential for good compliance make them suitable for pediatric populations. Furthermore, additional interventions, such as low-concentration atropine eye drops and defocus-incorporated multiple segment lenses, have demonstrated efficacy in reducing myopia progression (13–15). Notably, combining OK lenses with low-concentration atropine has shown potential synergistic effects, further enhancing AL control (16, 17). These complementary methods present opportunities for tailored treatment plans that can address individual patient needs (18). However, the effectiveness of OK lenses and other interventions varies considerably among patients due to factors such as age, baseline AL, and refractive error, highlighting the limitations of standardized treatment protocols that fail to account for individual differences (10).

Current management strategies for OK therapy often rely on fixed follow-up protocols, which overlook key patient-specific factors (11). As a result, clinicians face challenges in proactively addressing variable outcomes, such as rapid AL elongation or rebound effects following treatment discontinuation (19). Identifying high-risk patients, particularly younger children or those with rapid AL growth, is critical for optimizing follow-up schedules and implementing timely interventions (20, 21). Conversely, reducing unnecessary monitoring for low-risk patients can help conserve healthcare resources while maintaining efficiency (22). Recent research highlights the potential of predictive models that integrate multiple variables, including demographic, ocular, and environmental factors, to enhance risk stratification and treatment personalization (23–25). Such models could transform clinical practice by facilitating individualized management strategies and improving outcomes (26).

This study introduces a new approach to address the lack of predictive tools for individualizing OK therapy. It combines least absolute shrinkage and selection operator (LASSO) regression with logistic regression to identify key predictive variables. The primary target variable of our predictive model is the annual AL growth rate in children undergoing OK therapy, with a growth rate > 0.19 mm/year defined as an adverse outcome (27). A nomogram was created to visually display risk predictions, making it easier to apply in clinical settings. The model’s performance was validated using calibration plots, receiver operating characteristic (ROC) curves, and decision curve analysis (DCA) in both training and validation datasets. These steps ensure the model’s reliability and robustness. The model may provide information that clinicians could potentially use to identify high-risk patients early and generate individualized predictions of adverse outcomes. Based on these predictions, clinicians can then make informed decisions to optimize follow-up schedules and treatment strategies, addressing the current limitations of standardized protocols.

## Materials and methods

### Research design and study population

This study was a single-center retrospective cohort study conducted at Yuyao Maternity and Child Health Hospital. The data were collected from patient records between January 2015 and December 2021. The study included myopic patients who were fitted with OK lenses at the hospital. Ethical approval was obtained from the Ethics Committee of Yuyao Maternity and Child Health Hospital (Approval No. 2024YPT01), and all procedures complied with the Declaration of Helsinki. Written informed consent was obtained from all participants and their legal guardian(s).

Inclusion and Exclusion Criteria: Patients were included if they met the following criteria: (1) were aged 8–14 years; (2) had spherical equivalent refraction between −0.75D and −6.00D; (3) had astigmatism ≤ 2.00D; (4) had AL between 22.0 and 26.0 mm; (5) had intraocular pressure (IOP) < 21 mmHg; (6) had corneal curvature between 40.00D and 46.00D; and (7) had no history of wearing contact lenses or OK lenses previously. Patients were excluded if they: (1) had any ocular or systemic diseases; (2) their guardians refused follow-ups or phone inquiries; (3) had undergone other myopia treatments before OK lenses; (4) developed chronic diseases, tumors, or experienced severe trauma with unstable vital signs during treatment; or (5) had poor-quality corneal topography sampling, suboptimal lens fitting, or conditions such as color blindness or color weakness (11, 28, 29). During the study period, 527 patients were initially screened for eligibility. Eighty-three patients were excluded based on pre-specified criteria, including baseline parameters not meeting inclusion requirements (*n* = 30), guardian refusal of follow-up participation (*n* = 23), previous myopia control treatments (*n* = 18), and pre-existing diseases or poor examination quality (*n* = 12). The remaining 444 patients were enrolled and initiated OK lens treatment. During the first year of follow-up, five patients (1.1%) were lost to follow-up due to relocation (*n* = 2) and inability to maintain contact (*n* = 3). The final analysis included 439 patients who completed all required visits within the first year. The reported 292 adverse outcomes (66.5%) represent complete data from these 439 patients with complete follow-up data for all key variables.

### Lenses

All patients underwent standard anterior segment and refractive assessments. Cycloplegic refraction was performed using 1% tropicamide eye drops administered twice at 5-min intervals, with measurements taken 30 min after the first drop. Autorefraction was measured with the NIDEK AR-310A under cycloplegia. Baseline corneal topography was performed using the SW6000 topographer (ensuring corneal exposure ≥ 95% and a curvature control error within ± 0.25D). AL was measured using the IOL Master 500 (five repeated measurements averaged), and IOP was measured with the CT-800. Pupillary dilation and OCT were performed as needed to confirm fundus status. Lenses were prescribed based on corneal topography and refractive data, trial-fitted to ensure proper alignment, and then dispensed.

### Data collection

All patients were followed according to a standardized protocol, which included follow-up visits at 1 month, 3 months, 6 months, and 1 year after OK lenses fitting. After the first year, patients were followed every 6 months until the end of the study period. Patients were required to have at least these four follow-up visits to be included in the analysis. The maximum follow-up duration was set at 3 years. This study analyzed data from the right eye of each participant (30). All data were obtained through standardized equipment measurements and patient medical records, supplemented by telephone interviews when necessary. The collected variables included demographic information, clinical characteristics, and ocular parameters.

The demographic information consisted of age, sex, and parental myopia status. Clinical characteristics included the time spent outdoors (hours per day). Ocular parameters encompassed spherical refraction, cylindrical refraction, spherical equivalent refraction (SER), flat keratometry (Flat-K), steep keratometry (Steep-K), white-to-white distance (WTW), eccentricity, axial length (AL), intraocular pressure (IOP), astigmatism, and surface regularity index (SRI).

### Definition of adverse outcomes

The primary outcome of this study was the annual AL growth rate. An annual growth rate ≤ 0.19 mm was defined as a favorable outcome, while a growth rate > 0.19 mm was classified as an adverse outcome, this cutoff has been used in previous OK-related myopia control studies as a clinically meaningful threshold beyond which treatment effect is considered suboptimal (27, 31, 32). The annual AL growth rate was calculated by dividing the AL increase during the follow-up period by the number of follow-up months, then multiplying by 12. Based on this calculation, patients were categorized into the favorable outcome group or the adverse outcome group.

### Statistical analysis

All statistical analyses were performed using R software (version 4.4.1) and SPSS software (version 26.0), with a significance level set at *p* < 0.05. Continuous variables with a normal distribution were expressed as mean ± standard deviation and compared using independent *t*-tests. Non-normally distributed continuous variables were presented as median (interquartile range) and compared using Mann-Whitney U tests. Categorical variables were expressed as proportions and compared using chi-square tests.

The data were randomly divided into training and test sets in a 7:3 ratio using stratified splitting based on the primary outcome (favorable vs. adverse). This stratification technique ensured balanced distribution of outcome categories between the training and test sets, maintaining similar prevalence of adverse outcomes in both datasets and thereby enhancing model generalizability. The 7:3 ratio for training and test set allocation was selected to balance between having adequate data for model training (70%) while retaining sufficient cases for model validation (30%), which is widely used in predictive modeling research. Sample size determination was based on the generally accepted rule for predictive modeling that requires at least 10 events per predictor variable (33, 34). Our sample of 439 participants (with 292 adverse outcomes) provided sufficient statistical power for model development and validation.

In the training set, LASSO regression was applied for variable selection, using 10-fold cross-validation to determine the optimal penalty parameter (λ). L1 regularization was employed to retain the most relevant variables. Subsequently, a logistic regression model was constructed based on the selected variables, and a nomogram was developed to visually represent the predictive results. Model validation was conducted in both the training and test sets, including calibration plots, ROC curves, and DCA. The Hosmer-Lemeshow test was performed to evaluate model calibration. Calibration plots assessed the agreement between predicted probabilities and observed outcomes. ROC curves quantified the model’s discriminatory ability through the area under the curve (AUC). DCA evaluated the clinical utility of the model across various threshold probabilities.

## Results

### Baseline characteristics comparison

A total of 439 participants were included (308 in training set, 131 in test set). The baseline characteristics of the two groups are summarized in Table 1. The mean age was 10.85 ± 1.76 years, with 48.52% males. Parental myopia was common, with 53.99% having both parents myopic, 32.80% having one myopic parent, and 13.21% having no parental myopia. All demographic variables, including age and sex, showed no significant differences between training and test sets. Similarly, all ocular parameters including WTW, eccentricity, AL, IOP, astigmatism, and refractive measures were comparable between groups (all *p* > 0.05). The median follow-up time was 26.00 months (IQR: 24.00–29.00) for the entire cohort. Adverse outcomes occurred in 66.51% of participants, with similar rates in both training (66.56%) and test (66.41%) sets (*p* = 0.976). This balanced distribution confirms appropriate randomization for model development and validation.

**TABLE 1:** Baseline characteristics of participants in training and test sets.

Variables	Total (*n* = 439)	Training (*n* = 308)	Test (*n* = 131)	*t*/*Z*/χ^2^	*p*
Outcome, n(%)				0.00	0.976
Favorable outcome	147 (33.49)	103 (33.44)	44 (33.59)		
Adverse outcome	292 (66.51)	205 (66.56)	87 (66.41)		
Age, mean ± SD	10.85 ± 1.76	10.80 ± 1.75	10.98 ± 1.79	−0.95	0.341
Sex, n(%)				1.35	0.246
Male	213 (48.52)	155 (50.32)	58 (44.27)		
Female	226 (51.48)	153 (49.68)	73 (55.73)		
Parental myopia, n(%)				2.07	0.356
Neither myopic	58 (13.21)	45 (14.61)	13 (9.92)		
One myopic	144 (32.80)	97 (31.49)	47 (35.88)		
Both myopic	237 (53.99)	166 (53.90)	71 (54.20)		
Time spent outdoors (hours), M (Q1, Q3)	1.50 (1.00, 2.00)	1.50 (1.00, 2.00)	1.50 (1.00, 1.75)	−1.70	0.088
Flat K, Mean ± SD	42.90 ± 1.08	42.92 ± 1.10	42.87 ± 1.05	0.42	0.677
Steep K, Mean ± SD	43.96 ± 1.15	43.98 ± 1.15	43.91 ± 1.16	0.64	0.523
WTW, Mean ± SD	11.89 ± 0.28	11.89 ± 0.28	11.90 ± 0.29	−0.21	0.834
Eccentricity, mean ± SD	0.60 ± 0.08	0.60 ± 0.08	0.60 ± 0.09	−0.29	0.771
AL, mean ± SD	24.90 ± 0.68	24.89 ± 0.68	24.90 ± 0.69	−0.14	0.892
IOP, M (Q1, Q3)	16.00 (15.00, 19.00)	17.00 (15.00, 19.00)	16.00 (15.00, 19.00)	−0.12	0.905
Astigmatism, M (Q1, Q3)	1.02 (0.76, 1.30)	1.02 (0.77, 1.32)	1.02 (0.70, 1.25)	−0.47	0.640
Spherical refraction, M (Q1, Q3)	−2.75 (−3.75, −1.75)	−2.75 (−3.75, −1.94)	−2.75 (−3.75, −1.75)	−0.16	0.876
Cylindrical refraction, M (Q1, Q3)	−0.50 (−0.75, −0.25)	−0.50 (−0.75, −0.25)	−0.50 (−0.75, −0.25)	−0.39	0.698
SER, M (Q1, Q3)	−3.00 (−4.00, −2.12)	−3.00 (−4.00, −2.12)	−3.12 (−4.00, −2.12)	−0.09	0.927
SRI, M (Q1, Q3)	0.33 (0.32, 0.34)	0.33 (0.32, 0.34)	0.33 (0.32, 0.34)	−1.12	0.261
Follow−up time (months), M (Q1, Q3)	26.00 (24.00, 29.00)	26.00 (24.00, 29.00)	27.00 (23.00, 28.00)	−0.34	0.736

Flat K, flat corneal curvature; Steep K, steep corneal curvature; WTW, white-to-white distance; AL, axial length; IOP, intraocular pressure; SER, spherical equivalent refraction; SRI, surface regularity index.

### Baseline characteristics of favorable and adverse outcome groups in the training set

The baseline characteristics of the favorable and adverse outcome groups in the training set are presented in Table 2. Among the 308 patients in the training set, 103 (33.44%) had favorable outcomes and 205 (66.56%) had adverse outcomes. Significant age differences were observed between the groups, with patients in the favorable outcome group being older (11.81 ± 1.70 years) compared to those in the adverse outcome group (10.30 ± 1.55 years; *p* < 0.001).

**TABLE 2:** Baseline comparison between favorable and adverse outcome groups.

Variables	Total (*n* = 308)	Favorable outcome (*n* = 103)	Adverse outcome (*n* = 205)	*t*/*Z*/χ^2^	*p*
Age, mean ± SD	10.80 ± 1.75	11.81 ± 1.70	10.30 ± 1.55	7.80	< 0.001
Sex, n(%)				0.27	0.601
Male	155 (50.32)	54 (52.43)	101 (49.27)		
Female	153 (49.68)	49 (47.57)	104 (50.73)		
Parental myopia, n(%)				25.53	< 0.001
Neither myopic	45 (14.61)	27 (26.21)	18 (8.78)		
One myopic	97 (31.49)	39 (37.86)	58 (28.29)		
Both myopic	166 (53.90)	37 (35.92)	129 (62.93)		
Time spent outdoors (hours), M (Q1, Q3)	1.50 (1.00, 2.00)	1.50 (1.00, 2.00)	1.50 (1.00, 2.00)	−0.53	0.594
Flat K, Mean ± SD	42.92 ± 1.10	42.94 ± 1.04	42.90 ± 1.13	0.26	0.797
Steep K, mean ± SD	43.98 ± 1.15	44.06 ± 1.04	43.94 ± 1.21	0.91	0.366
WTW, Mean ± SD	11.89 ± 0.28	11.94 ± 0.29	11.87 ± 0.28	2.24	0.026
Eccentricity, mean ± SD	0.60 ± 0.08	0.59 ± 0.07	0.60 ± 0.08	−1.30	0.194
AL, mean ± SD	24.89 ± 0.68	25.07 ± 0.66	24.80 ± 0.67	3.34	< 0.001
IOP, M (Q1, Q3)	17.00 (15.00, 19.00)	16.00 (15.00, 19.00)	17.00 (15.00, 18.00)	−0.47	0.636
Astigmatism, M (Q1, Q3)	1.02 (0.77, 1.32)	1.03 (0.80, 1.34)	1.02 (0.76, 1.32)	−0.19	0.846
Spherical refraction, M (Q1, Q3)	−2.75 (−3.75, −1.94)	−3.25 (−4.25, −2.25)	−2.50 (−3.50, −1.75)	−4.25	< 0.001
Cylindrical refraction, M (Q1, Q3)	−0.50 (−0.75, −0.25)	−0.50 (−0.75, −0.25)	−0.50 (−0.75, −0.25)	−1.27	0.203
SER, M (Q1, Q3)	−3.00 (−4.00, −2.12)	−3.50 (−4.50, −2.62)	−2.75 (−3.75, −2.00)	−4.03	< 0.001
SRI, M (Q1, Q3)	0.33 (0.32, 0.34)	0.33 (0.32, 0.34)	0.33 (0.32, 0.34)	−0.98	0.329
Follow-up time (months), M (Q1, Q3)	26.00 (24.00, 29.00)	26.00 (24.00, 28.00)	27.00 (24.00, 29.00)	−0.47	0.635

Parental myopia status showed significant differences between the groups (*p* < 0.001). In the favorable outcome group, 26.21% had no myopic parents, 37.86% had one myopic parent, and 35.92% had both parents with myopia. In contrast, the adverse outcome group showed a higher proportion of patients with both myopic parents (62.93%) and lower rates of non-myopic parents (8.78%).

Regarding ocular parameters, mean AL was significantly longer in the favorable outcome group (25.07 ± 0.66 mm) compared to the adverse outcome group (24.80 ± 0.67 mm; *p* < 0.001). WTW was also significantly greater in the favorable outcome group (11.94 ± 0.29 mm vs. 11.87 ± 0.28 mm; *p* = 0.026).

Refractive parameters showed significant differences, with the favorable outcome group having more myopic spherical refraction (median: −3.25 D vs. −2.50 D; *p* < 0.001) and SER (median: −3.50 D vs. −2.75 D; *p* < 0.001).

No significant differences were observed between the groups in terms of gender distribution, time spent outdoors, keratometry values, eccentricity, IOP, astigmatism, cylindrical refraction, SRI, or follow-up duration (all *p* > 0.05).

### LASSO variable selection

**FIGURE 1:**
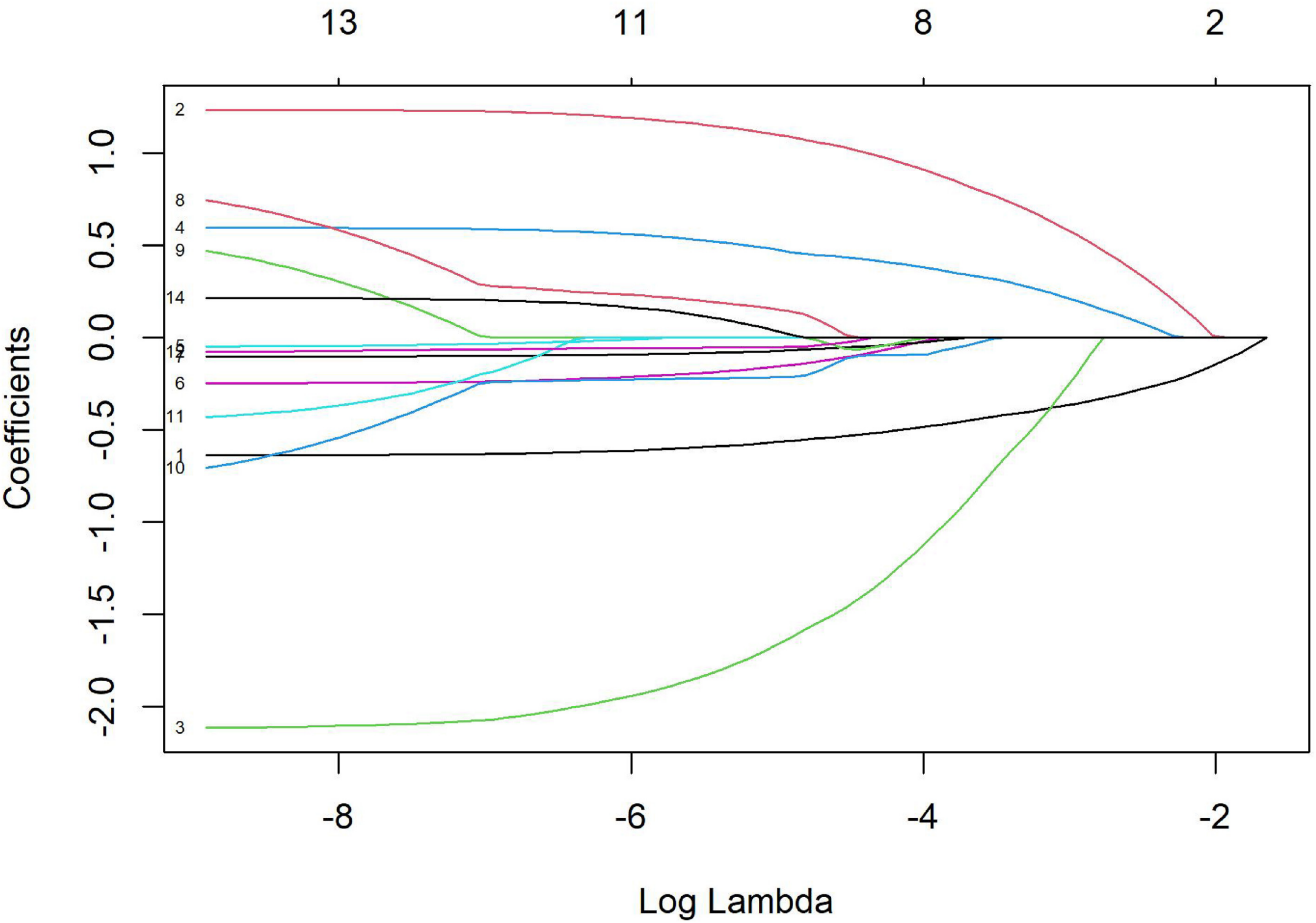
LASSO coefficient path for variable selection.

**FIGURE 2:**
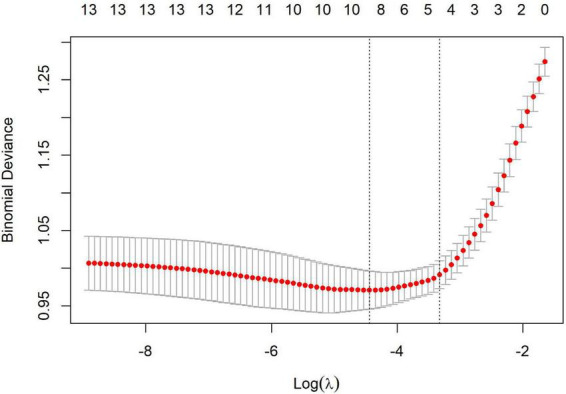
Cross-validation curve for optimal lambda selection in LASSO.

The results of the LASSO regression are illustrated in Figures 1, 2. Variable selection was performed using LASSO regression with 10-fold cross-validation to determine the optimal penalty parameter (λ). Figure 1 displays the coefficient path of each variable as λ changes, where each colored line represents a different predictor variable, and the numbers at the top of the plot indicate the number of non-zero coefficients at each λ value. As the regularization parameter λ increases (moving from left to right), the coefficient trajectories show how variables are progressively eliminated with increasing regularization, with coefficients shrinking toward zero. Figure 2 presents the cross-validation curve showing the relationship between binomial deviance (mean-squared error) and log(λ), where red dots represent the cross-validation error at each λ value, error bars indicate the standard error, and the numbers at the top denote the number of variables included in the model at each point. The two vertical dashed lines indicate λ.min (left) representing the λ value with minimum cross-validation error, and λ0.1se (right) representing the λ value within one standard error of the minimum.

We carefully evaluated both λ.min (minimum mean cross-validated error) and λ0.1se (one standard error rule) approaches to determine the most appropriate model. While λ.min (λ = 0.012) initially identified nine variables (age, parental myopia history, baseline WTW, baseline spherical refraction, outdoor activity time, baseline IOP, baseline flat keratometry, baseline steep keratometry, and baseline cylindrical refraction), we ultimately selected the λ0.1se approach (λ = 0.036), which retained only four key variables: age, parental myopia history, baseline WTW, and baseline spherical refraction.

This decision was guided by model parsimony principles and information criteria comparisons. The Akaike Information Criterion (AIC), which estimates prediction error by balancing goodness of fit against model complexity, was 297.63 for the reduced model versus 299.14 for the full model. Similarly, the Bayesian Information Criterion (BIC), which imposes a more stringent penalty for additional parameters than AIC, was 320.01 for the reduced model compared to 340.17 for the full model. The lower values for both AIC and BIC in the reduced model indicate superior performance when accounting for model complexity. Furthermore, when we applied optimal subset selection to the full nine-variable model, we obtained the same four variables, further validating our approach. This convergence of selection methods reinforces the importance of these four variables as the most significant predictors while minimizing redundancy and multicollinearity in the final model.

### Logistic regression analysis and nomogram

The variables selected through LASSO regression were subjected to logistic regression analysis, with the results presented in Table 3. Age emerged as a significant protective factor against adverse outcomes (OR = 0.552, 95% CI: 0.458–0.667, *p* < 0.001), indicating that older patients had substantially reduced risk of adverse outcomes. Parental myopia demonstrated a strong dose-dependent relationship with myopia progression, with both one myopic parent (OR = 5.428, 95% CI: 2.184–13.491, *p* < 0.001) and two myopic parents (OR = 12.462, 95% CI: 5.098–30.463, *p* < 0.001) significantly increasing the risk of adverse outcomes compared to children with no parental myopia.

**TABLE 3:** Multivariable logistic regression analysis for predicting adverse outcomes.

Variables	β	S.E.	Wald	*p*	OR (95%CI)
Age	−0.593	0.096	38.185	< 0.001	0.552 (0.458, 0.667)
**Parental myopia (ref = Neither myopic)**					
One myopic	1.692	0.465	13.259	< 0.001	5.428 (2.184, 13.491)
Both myopic	2.523	0.456	30.603	< 0.001	12.462 (5.098, 30.463)
WTW	−1.605	0.523	9.408	0.002	0.201 (0.072, 0.560)
Spherical refraction	0.529	0.136	15.183	< 0.001	1.697 (1.301, 2.214)

WTW was identified as another protective factor (OR = 0.201, 95% CI: 0.072–0.560, *p* = 0.002), with larger WTW associated with a markedly lower risk of adverse outcomes. Conversely, spherical refraction was a significant risk factor (OR = 1.697, 95% CI: 1.301–2.214, *p* < 0.001), with higher (less negative) refraction values associated with increased risk of adverse outcomes.

**FIGURE 3:**
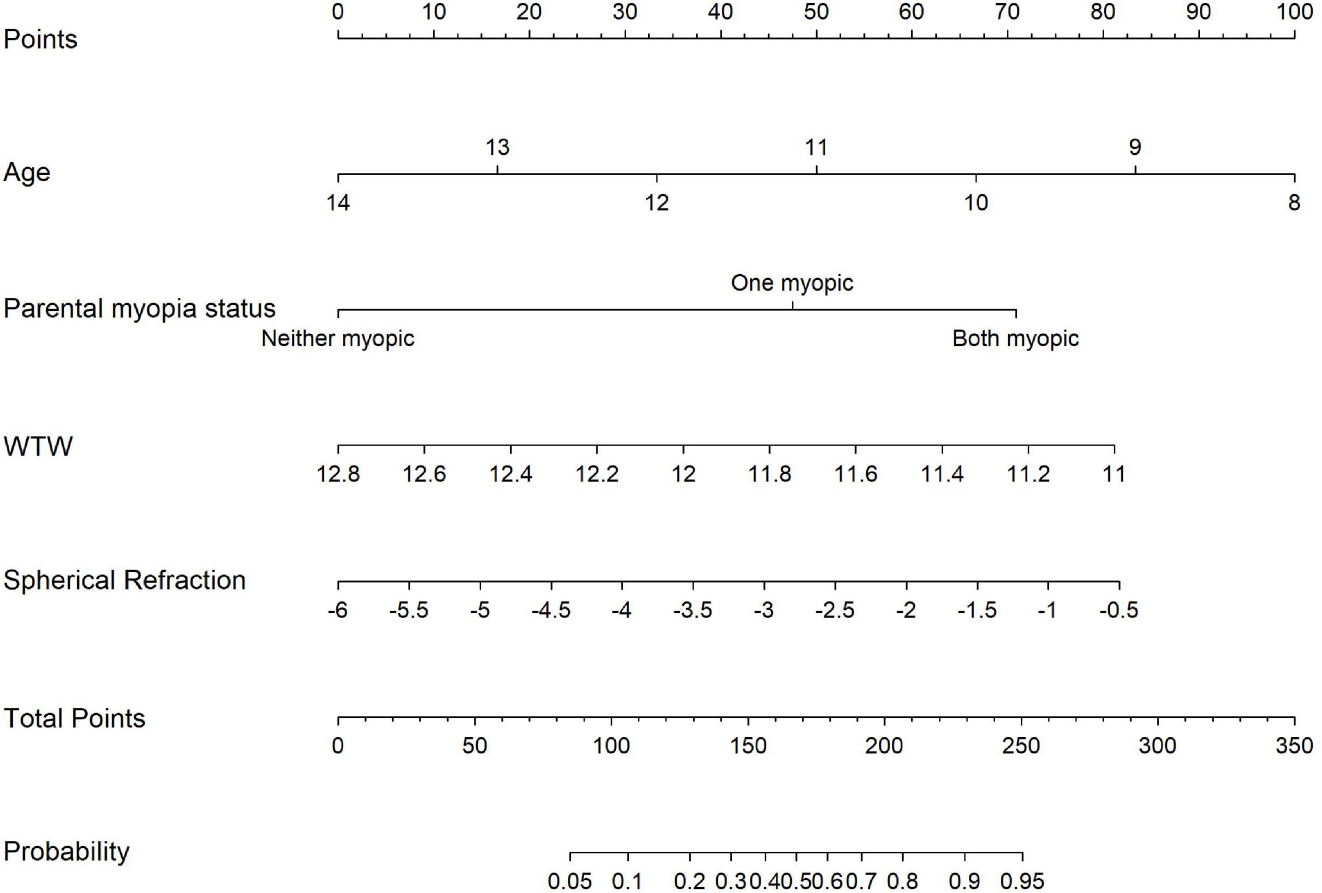
Nomogram for predicting the risk of adverse outcomes.

Based on the logistic regression model, a nomogram (Figure 3) was constructed to predict individual risk of adverse outcomes in children undergoing OK therapy. The nomogram integrates all predictive variables identified in the analysis. Each variable corresponds to a specific point value on the nomogram, and the total points, calculated by summing the individual variable points, correlates with the predicted probability of adverse outcomes.

### Model validation

The predictive model was validated using calibration plots, ROC curves, and DCA in both the training and test sets.

**FIGURE 4:**
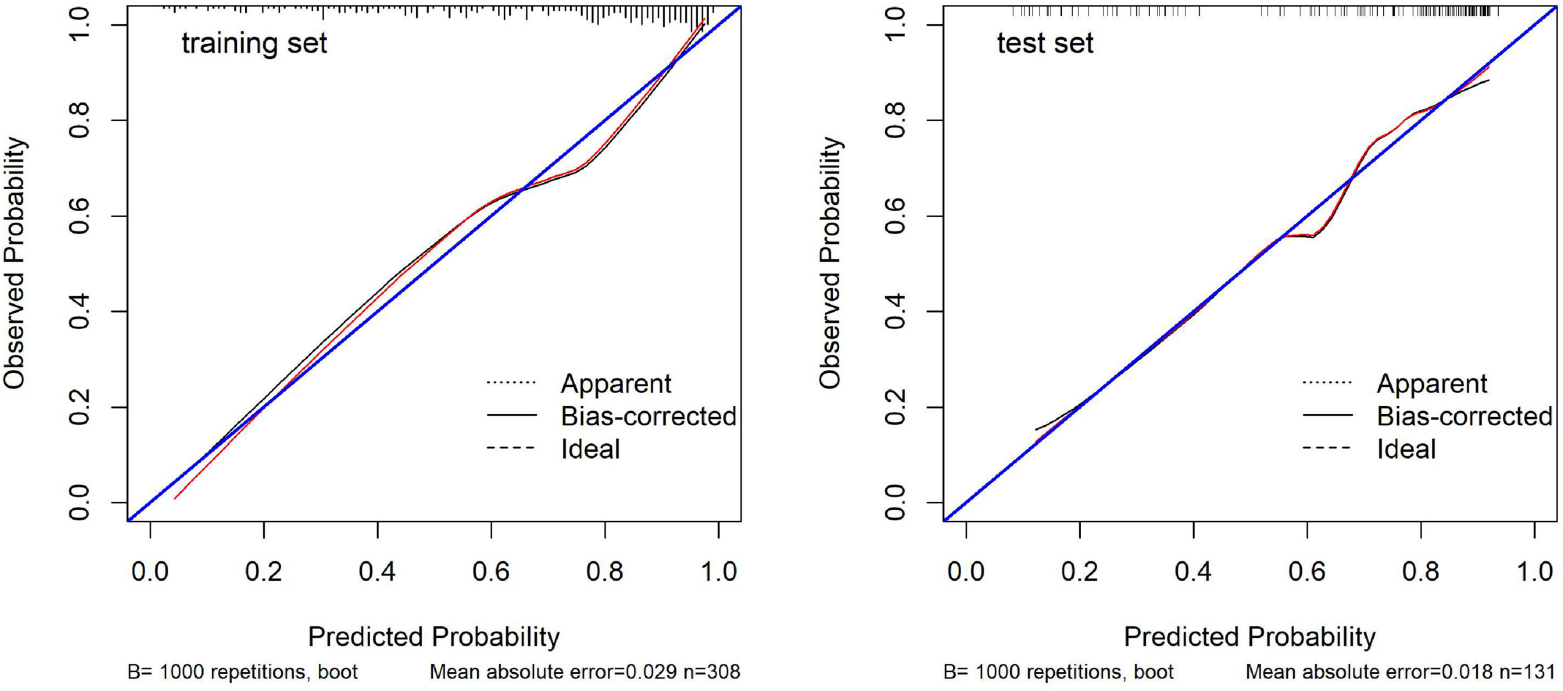
Calibration plots for the predictive model in the training and test sets.

Calibration plots (Figure 4) were used to assess the agreement between predicted probabilities and observed outcomes. These plots showed good calibration in both the training and test sets, with the calibration curves following the ideal diagonal line reasonably well. The Hosmer-Lemeshow test supported these findings, with non-significant results in both the training set (*p* = 0.504) and the test set (*p* = 0.863), indicating adequate model calibration.

**FIGURE 5:**
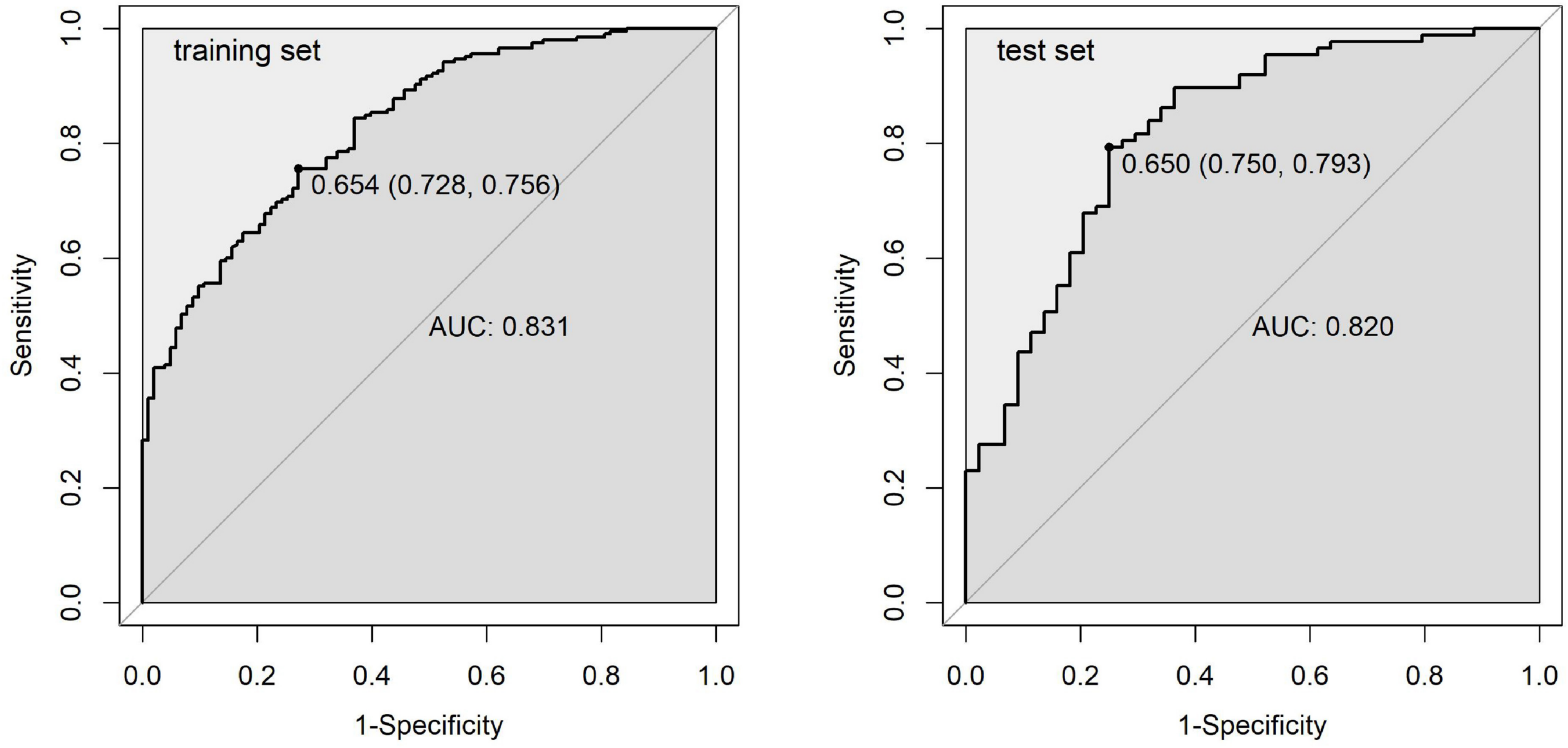
ROC curves for the predictive model in the training and test sets.

ROC curves (Figure 5) were used to evaluate the discriminatory ability of the predictive model. The area under the curve (AUC) was 0.831 (95% CI: 0.786–0.877) for the training set and 0.820 (95% CI: 0.742–0.899) for the test set, suggesting good predictive performance. Sensitivity and specificity metrics of 75.6 and 72.8% in the training set, and 79.3 and 75.0% in the validation set further support the model’s ability to correctly classify both positive and negative outcomes. These values indicate that the model has reasonable ability to distinguish between favorable and adverse outcomes. The similar performance across both datasets suggests that the model maintains its predictive capability when applied to new data.

**FIGURE 6:**
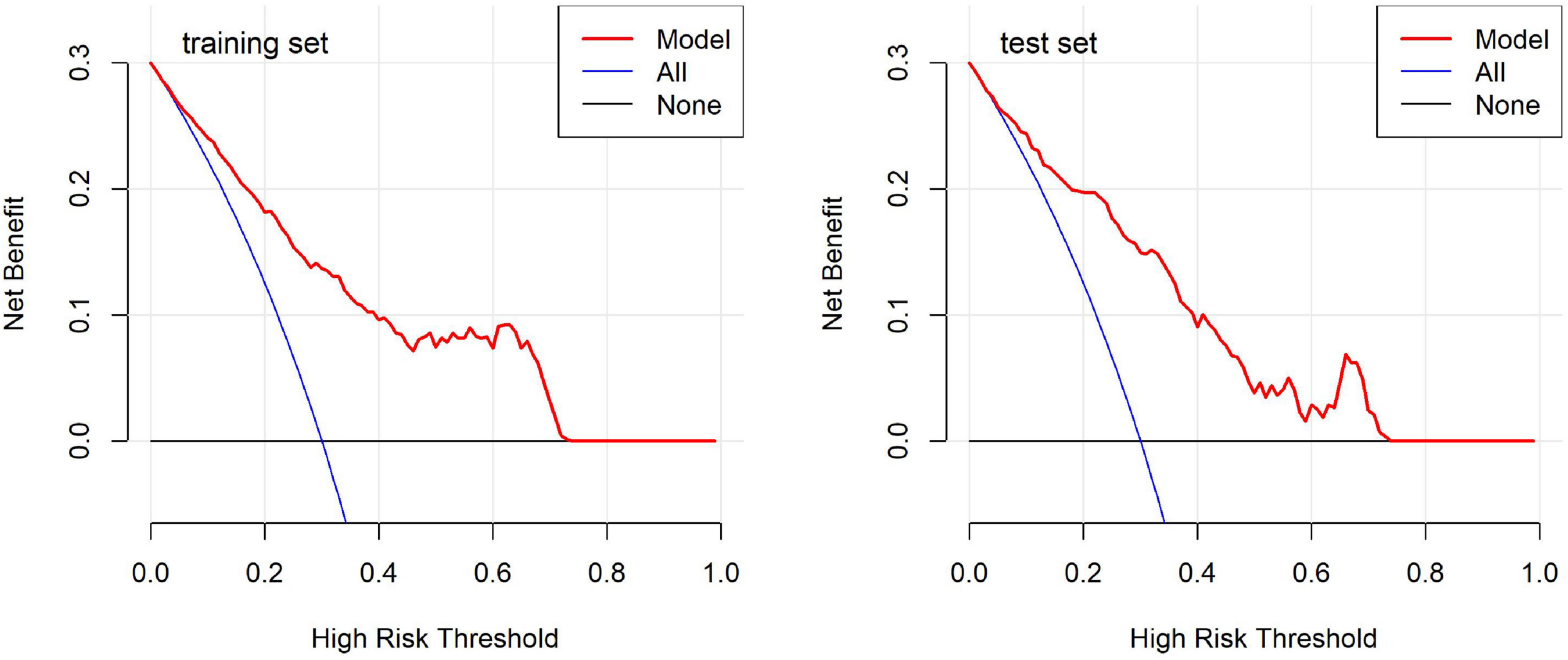
Decision curve analysis for the predictive model in the training and test sets.

DCA curves (Figure 6) evaluated the clinical utility of the model across various threshold probabilities. The results showed that the net benefit curve of the model generally exceeded the “treat-all” and “treat-none” baselines across a range of clinically relevant threshold probabilities in both datasets. This suggests potential clinical value in using the model to guide decision-making regarding monitoring and intervention strategies for children undergoing OK therapy. Collectively, these validation results indicate that our predictive model demonstrates promising discriminatory ability, satisfactory calibration, and potential clinical utility, which may contribute to risk stratification approaches in pediatric myopia management.

## Discussion

This study attempted to develop and validate a predictive model for OK therapy outcomes, which showed promising discriminatory ability in our sample. The model achieved an AUC of 0.831 in the training set and 0.820 in the validation set, consistent with another study that reported an AUC around 0.8 (35). Sensitivity and specificity metrics of 75.6 and 72.8% in the training set, and 79.3 and 75.0% in the validation set, further confirm the model’s reliability across datasets. These preliminary results suggest the model might have potential for clinical applications, though further validation is necessary before implementation in myopia management approaches.

The random splitting of the data into training and validation sets was effective, this balanced split ensures that the model’s performance is not biased by uneven distributions of key predictors, enhancing its generalizability (36). Table 2 provides insights into the differences between favorable and adverse outcome groups within the training set. Significant differences in variables such as baseline AL, spherical refraction, and SER highlight their relevance to myopia progression. However, given the strong correlation among these variables, LASSO regression was employed to select the most predictive and non-redundant variables. By determining the optimal λ value, LASSO regression attempted to address multicollinearity, suggesting four variables that may have value in forecasting adverse outcomes (37).

The exclusion of five variables (outdoor activity time, baseline IOP, flat keratometry, steep keratometry, and cylindrical refraction) at λ0.1se warrants discussion. The λ0.1se approach applies stricter regularization to eliminate variables with weaker predictive power or redundancy with retained predictors. Outdoor activity time may have been excluded due to collinearity with age, as behavioral patterns vary across age groups in pediatric populations. The exclusion of both corneal keratometry measurements while retaining WTW likely reflects redundancy among these corneal anatomical parameters. Baseline IOP and cylindrical refraction likely demonstrated modest independent effect sizes. This interpretation is supported by our model comparison results, where the four-variable model showed superior performance metrics (AIC = 297.63, BIC = 320.01) compared to the nine-variable model (AIC = 299.14, BIC = 340.17). These patterns align with LASSO’s statistical properties, which eliminate redundant or weakly predictive variables while preserving those with the strongest independent associations with the outcome. The convergence with optimal subset selection further validates this approach.

Our findings underscore the multifactorial nature of myopia progression and the complex interplay between various predictors. Age emerged as a significant protective factor, with older children showing reduced risk of adverse outcomes. This aligns with established understanding that younger children experience faster AL elongation due to active ocular growth (30). The protective role of age in our model may be attributed to several factors. Firstly, the rate of eye growth tends to slow down as children approach adolescence, which could contribute to better OK therapy outcomes in older children (38). Secondly, older children may have better compliance with lens wear and care instructions, potentially enhancing treatment efficacy.

Peripheral retinal mechanisms play a crucial role in emmetropization and myopia development. While the direct relationship between WTW distance and OK therapy outcomes is not yet fully established, corneal parameters have been shown to influence OK lenses fitting and centration (39, 40). The WTW distance, as a measure of corneal diameter, may affect the mechanical interaction between the lens and corneal surface, thereby potentially influencing lens positioning and the resulting treatment zone characteristics. Studies have demonstrated that OK lenses induce changes in peripheral refraction, which may contribute to myopia control effects (41). These findings contribute to our understanding of the factors influencing AL growth, which is critical in modulating myopia progression (42).

We found that children with milder myopia at baseline may be at higher risk of faster AL elongation. This observation aligns with previous research, which reported that subjects with lower initial myopia showed greater axial elongation over 2 years of OK treatment (43). Further studies have supported this relationship between initial refraction and myopia progression rate in OK therapy (11). Children with lower initial myopia experienced faster axial elongation during OK treatment compared to those with higher initial myopia. While this observation might initially appear unexpected, it is important to note that children with milder myopia in our cohort tended to be younger, and age emerged as a significant protective factor in our model. The faster axial elongation in children with milder myopia may therefore be partly attributable to their younger age and the associated more active phase of ocular growth. Nevertheless, this finding emphasizes the need for careful monitoring and potentially more aggressive treatment strategies in cases of mild myopia, particularly in younger children.

The inclusion of parental myopia history as a significant risk factor highlights the genetic predisposition to myopia progression. Our findings revealed a notable dose-dependent relationship, with children having both myopic parents demonstrating substantially higher risk compared to children with no parental myopia. Similarly, children with only one myopic parent showed significantly increased risk compared to those with no parental myopia. This finding corroborates previous research on the heritability of refractive errors and suggests that genetic factors may influence not only the onset of myopia but also its progression rate during OK therapy (44). The strong association with parental myopia in our model emphasizes the importance of considering family history when assessing risk profiles for myopia progression in children undergoing OK therapy.

From a clinical perspective, our predictive model may potentially serve as a tool for tailoring interventions based on individualized risk profiles, though clinical validation is still required. The nomogram may help clinicians identify patients who could benefit from more intensive monitoring and potential adjunctive treatments alongside OK therapy (45, 46). For example, a younger child with mild myopia, smaller WTW, and two myopic parents would likely have higher risk scores, suggesting more careful follow-up may be warranted. Conversely, an older adolescent with larger WTW and no family history of myopia might require less intensive monitoring. This approach could potentially optimize both patient care and resource allocation in clinical settings by directing more attention to higher-risk individuals while reducing unnecessary follow-ups for those at lower risk (47). The notable influence of parental myopia in our model suggests that family history assessment should be considered when developing monitoring strategies. This may be particularly relevant in regions with high myopia prevalence, where efficient allocation of clinical resources is important. The incorporation of our predictive model into clinical practice may help enhance the management of pediatric myopia. By offering a quantitative assessment of individual risk, clinicians could potentially have more informed discussions with patients and their families about the possible outcomes of OK therapy (7). This more personalized approach might contribute to treatment adherence and patient satisfaction through better expectation management. The model may also assist in identifying higher-risk individuals earlier in the treatment process, which could allow for more timely adjustments to management strategies. While further validation is needed, such approaches represent a step toward more individualized care in myopia management.

Our predictive model may also inform treatment selection among myopia control modalities. Orthokeratology slows myopia through peripheral myopic defocus (41). Alternative approaches include low-concentration atropine, which acts via muscarinic receptor pathways (48), and repeated low-level red-light therapy, which increases choroidal thickness through photobiomodulation (49). High-risk patients identified by our model might benefit from combination therapies or alternative interventions tailored to individual risk profiles.

Our findings also have implications for the design of future clinical trials in myopia control. The identification of key predictive factors can inform patient selection criteria and stratification strategies in randomized controlled trials, potentially leading to more efficient and targeted studies. For example, future trials could focus on evaluating the efficacy of combination therapies in high-risk individuals identified by our model, or investigate novel interventions specifically tailored to patients with certain risk profiles (50). The role of corneal parameters in our model, particularly the WTW distance, highlights the importance of considering anterior segment characteristics in myopia management. While much of the recent focus in myopia research has been on retinal and choroidal factors, our findings suggest that corneal biomechanics and topography play a crucial role in determining OK therapy outcomes (51). This underscores the need for comprehensive anterior segment evaluation in myopic children, not only for OK lenses fitting but also for predicting treatment response. Our model’s performance in predicting OK therapy outcomes raises interesting questions about the underlying mechanisms of myopia progression and the mode of action of OK lenses. The complex interplay between demographic, ocular, and genetic factors in determining treatment outcomes suggests that myopia progression is not a uniform process across all individuals (52). Instead, it appears to be influenced by a combination of eye growth patterns, corneal biomechanics, and possibly systemic factors that are yet to be fully elucidated (53).

From a public health perspective, our model holds promise for integration into large-scale screening programs. By stratifying patients by risk, community health initiatives can prioritize high-risk groups for intensive interventions, potentially reducing long-term complications and associated healthcare costs. For example, school-based screening programs could incorporate this model to identify children at greatest risk, guiding resource allocation and treatment decisions at a population level. This approach could be particularly impactful in regions with limited healthcare resources, allowing for more efficient targeting of myopia control interventions.

The development of our predictive model also contributes to the broader field of personalized medicine in ophthalmology. As we move toward more individualized treatment approaches, predictive models like ours will play a crucial role in tailoring interventions to patient-specific factors (54). This shift toward precision medicine in myopia management aligns with similar trends in other areas of ophthalmology, such as glaucoma and age-related macular degeneration, where risk prediction models are increasingly being used to guide treatment decisions (55, 56). In the context of the growing global prevalence of myopia, our predictive model represents a significant step toward more effective and efficient myopia management strategies. If further validated, such models might contribute to the earlier identification of high-risk individuals and potentially assist in personalizing treatment approaches, which could help address the long-term burden of myopia-related complications. As research in this field continues to evolve, integration of additional factors such as environmental variables, detailed genetic profiles, and advanced imaging biomarkers could further enhance the predictive power and clinical utility of such models.

## Limitations

While our study provides valuable insights into predicting OK therapy outcomes, several limitations should be acknowledged. The single-center, retrospective design introduces potential center-specific bias and inherent risks of missing data and selection bias, which may limit generalizability. The model was validated using an internal test set rather than an independent external cohort, limiting assessment of its performance across diverse populations. Future multi-center prospective studies with external validation are warranted to confirm the model’s generalizability. The relatively short follow-up period restricts our ability to assess long-term outcomes. Although we included key demographic and ocular parameters, environmental factors and detailed genetic information were not fully explored. Future research incorporating these additional variables and external validation cohorts could further enhance the model’s predictive power and clinical utility.

## Conclusion

This study proposed a predictive model for OK therapy outcomes in myopic children with promising initial validation results. By integrating multiple patient-specific factors, including age, ocular parameters, and family history, the model showed encouraging discriminatory ability in identifying patients at risk of adverse outcomes. The nomogram may offer clinicians a potential tool for individualized risk assessment and treatment planning. This approach to myopia management, if validated in larger prospective studies, might contribute to optimizing patient care, resource allocation, and potentially enhance myopia control strategies.

## Data Availability

The raw data supporting the conclusions of this article will be made available by the authors, without undue reservation.
